# The reliability of shear elastic modulus measurement of the ankle plantar flexion muscles is higher at dorsiflexed position of the ankle

**DOI:** 10.1186/s13047-017-0199-0

**Published:** 2017-04-18

**Authors:** Junya Saeki, Tome Ikezoe, Masatoshi Nakamura, Satoru Nishishita, Noriaki Ichihashi

**Affiliations:** 1 Human Health Sciences, Graduate School of Medicine, Kyoto University, 53 Shogoin-Kawahara-cho, Sakyo-ku, Kyoto, 606-8507 Japan; 2 Research Fellow of the Japan Society for the Promotion of Science, 5-3-1 Kojimachi, Chiyoda-ku, Tokyo, 102-0083 Japan; 3 Institute for Human Movement and Medical Sciences, Niigata University of Health and Welfare, Shimami-cho 1398, Kita-ku, Niigata, 950-3198 Japan; 4 Institute of Rehabilitation Science, Tokuyukai Medical Corporation, 3-11-1 Sakurano-cho, Toyonaka, Osaka 560-0054 Japan; 5 Kansai Rehabilitation Hospital, 3-11-1 Sakurano-cho, Toyonaka, Osaka 560-0054 Japan

**Keywords:** Shear-wave elastography, Shear elastic modulus, Muscle stiffness, Muscle hardness, Reliability, Tibialis posterior, Flexor digitorum longus, Flexor hallucis longus

## Abstract

**Background:**

Excessive stiffness of lower limb muscles is associated with sports injuries. Therefore, the identification of a reliable measurement of the shear elastic modulus of various ankle plantar flexion muscles is required to evaluate lower leg sports injuries. This study investigated the reliable measurement of the shear elastic modulus of the ankle plantar flexion muscles at different ankle positions.

**Methods:**

Twenty-three healthy young men (25.3 ± 3.6 years, 172.9 ± 5.0 cm, 67.2 ± 7.2 kg) participated in this study. The shear elastic moduli of the ankle plantar flexion muscles including the lateral gastrocnemius, medial gastrocnemius, soleus, peroneus longus, peroneus brevis, flexor hallucis longus, flexor digitorum longus and tibialis posterior were measured using ultrasonic shear wave elastography at 0, 10 and 20° dorsiflexion.

**Results:**

The reliability of the shear elastic modulus measurements for each ankle position was assessed. The results showed that the interday reliability of the measurements differed between ankle positions and that the reliability of the shear elastic modulus measurements at 20° dorsiflexion was higher than that at 10° or 0°.

**Conclusion:**

Our results suggest that measurements at 20° dorsiflexion may provide a more reliable measurement of the shear elastic modulus of ankle plantar flexion muscles.

## Background

It has been reported that an overuse injury in sports is associated with soft tissue stiffness [1, 2]. In particular, the elongational stress of lower limb muscles such as the soleus (SOL), flexor digitorum longus (FDL) or tibialis posterior (TP) increases the strain in the tibial fascia [3]. These muscles have been identified as risk factors for medial tibial stress syndrome (MTSS), which commonly develops in runners. In addition, it is considered that excessive stiffness of the lower limb muscles is also associated with MTSS [4]. Therefore, the assessment and intervention of lower limb muscle stiffness are important for preventing MTSS.

In recent years, it has become possible to evaluate muscle stiffness quantitatively in vivo with the shear elastic modulus, which is calculated from the velocity of the shear wave created by the vibration of tissue using shear wave elastography [5–7]. Regarding the shear elastic modulus of the medial gastrocnemius (MG), lateral gastrocnemius (LG) and SOL using shear wave elastography, previous studies have shown its availability as an index of muscle stiffness [4, 8]. Other previous studies also demonstrated the reliability of shear elastic modulus measurements of the MG, LG and SOL [9, 10]. Although the shear elastic modulus of the triceps surae has been considered as a useful index of muscle stiffness, the reliability of shear elastic modulus measurements of other ankle plantar flexion muscles remains unclear. However, the muscle stiffnesses of the LG, MG, SOL and peroneus longus (PL) were higher in patients with MTSS [4]. Therefore, the identification of a reliable measurement of the shear elastic modulus of various ankle plantar flexion muscles is required to evaluate lower leg sports injuries. The purpose of this study was to investigate the reliability of the shear elastic modulus of the ankle plantar flexion muscles at different ankle positions and to determine the ankle position that can provide the most reliable measurement.

## Methods

### Subjects

Twenty-three healthy young men participated in this study. The mean values (± the standard deviation (SD)) for their age, height and mass were 25.3 ± 3.6 years, 172.9 ± 5.0 cm and 67.2 ± 7.2 kg, respectively. Subjects with a history of neuromuscular disease or musculoskeletal injury on the lower limbs were excluded. All participants had a maximum ankle dorsiflexion (DF) angle of at least 20°. Participants were given precise information about the content and order of the study. In addition, informed consent was obtained from all participants. This study was approved by the ethics committee of Kyoto University Graduate School and the Faculty of Medicine (R0266).

### Procedures

#### Measurements of shear elastic modulus

The shear elastic modulus of a muscle was measured using ultrasonic shear wave elastography (Aixplorer, Supersonic Imagine, France) with a linear array probe (SL10-2, Supersonic Imagine, France). The region of interest (ROI) was set near the centre of each muscle. Adopting the method used in a previous study [11], the shear elastic modulus μ was calculated as follows:

$$ \mu (kPa)=\rho V{s}^2, $$

where Vs is the shear wave velocity, and ρ is the muscle mass density (1000 kg · m^−3^). The analysis area was a 5-mm-diameter circle at the centre of the ROI [12]. The shear elastic modulus was measured three times per muscle, and the average of the three measured values was used for further analysis. To assess the reliability, the same protocol was performed on two different days by the same investigator at an interval of two weeks. The measurement of shear elastic modulus was performed by the same observer (JS) with at least 100 h of experience in ultrasound elastography. Considering the intraday fluctuation in the shear elastic modulus caused by muscle fatigue, the time of measurement for the interday analysis was matched as much as possible.

**Fig. 1:**
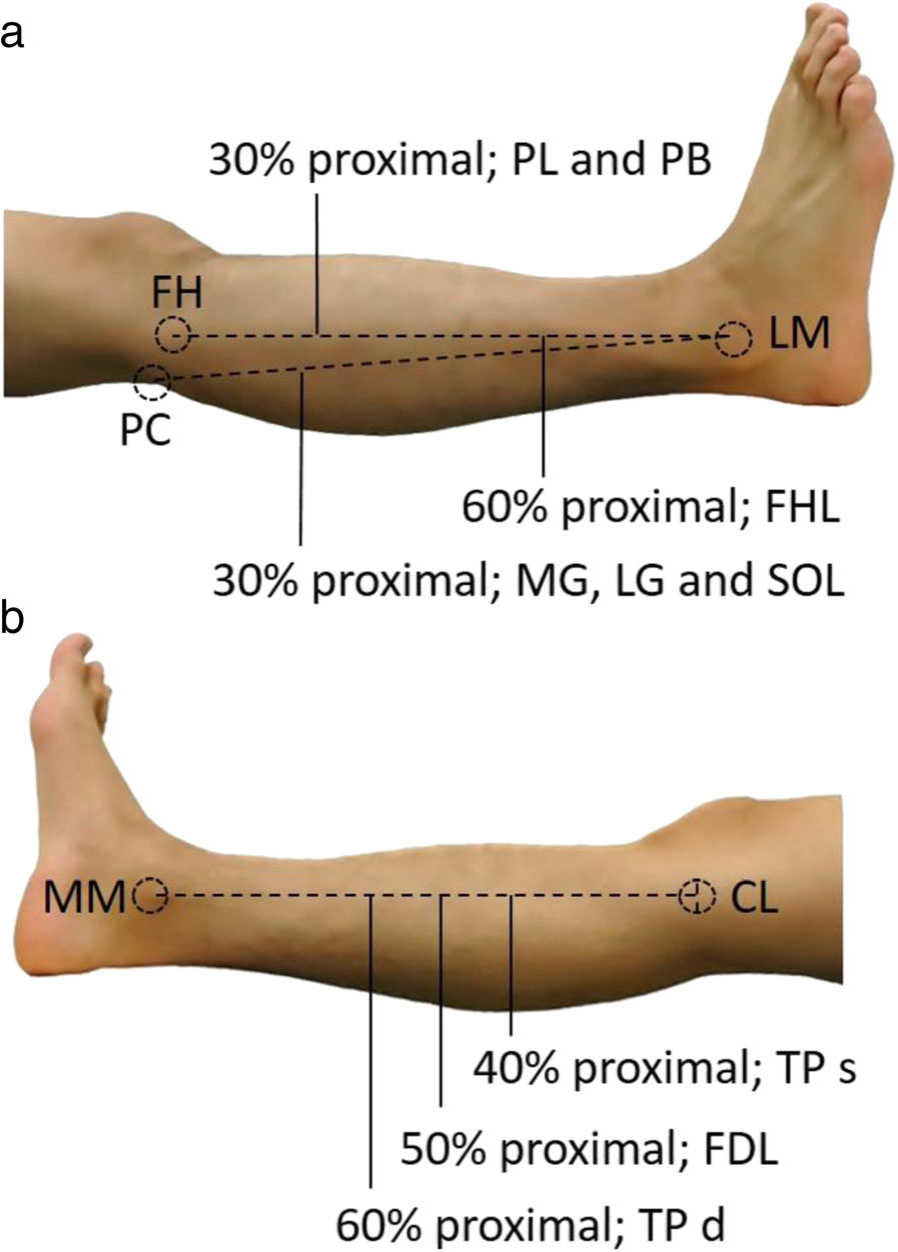
Locations of the shear elastic modulus measurement. (**a**) lateral (LG: lateral gastrocnemius; MG: medial gastrocnemius; SOL: soleus; PL: peroneus longus; PB: peroneus brevis; FHL: flexor halluces longus; PC: popliteal crease; FH: head of the fibula; LM: lateral malleolus) and (**b**) medial (FDL: flexor digitorum longus; TP s: tibialis posterior “superficial layer of intramuscular tendon”; TP d: tibialis posterior “deep layer of intramuscular tendon”; CL: cleavage line of the knee joint; MM: medial malleolus) sides

**Fig. 2:**
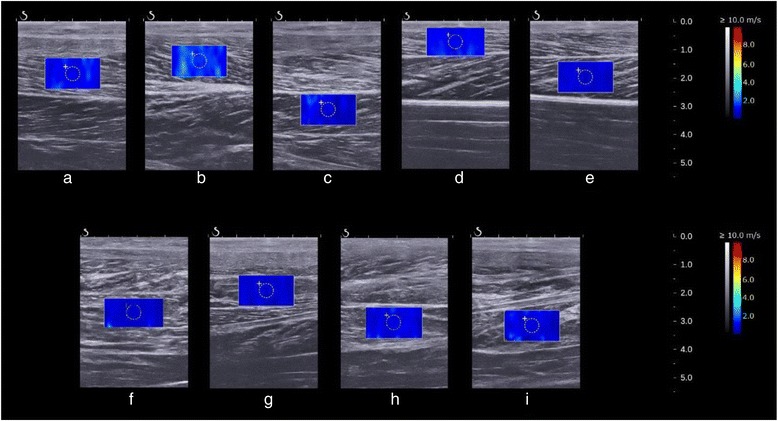
Ultrasound images of shear elastic modulus measurement. Ultrasound images of the shear elastic modulus measurement (**a**) lateral gastrocnemius (**b**) medial gastrocnemius (**c**) soleus (**d**) peroneus longus (**e**) peroneus brevis (**f**) flexor hallucis longus (**g**) flexor digitorum longus (**h**) tibialis posterior (superficial layer of intramuscular tendon) (**i**) tibialis posterior (deep layer of intramuscular tendon)

The target muscles were MG, LG, SOL, PL, peroneus brevis (PB), flexor halluces longus (FHL), FDL, and TP. These muscles have the moment arm of plantar flexion of the ankle [13]. The shear elastic moduli of the LG, MG, SOL, PL, PB, and FHL were measured in a prone position, while those for the FDL and TP were measured in a supine position. The shear elastic moduli of the LG, MG and SOL were measured at proximal 30% of the lower leg length from the popliteal crease to the lateral malleolus [8, 14]. The shear elastic moduli of the PL and PB were measured at proximal 30% from the head of the fibula to the lateral malleolus. The shear elastic modulus of the FHL was measured at proximal 60% from the head of the fibula to the lateral malleolus. The shear elastic modulus of the FDL was measured at proximal 50% from the cleavage line of the knee joint to the medial malleolus. The shear elastic modulus of the TP was measured at proximal 40% from the cleavage line of the knee joint to the medial malleolus for the superficial layer of the intramuscular tendon and at proximal 60% from the cleavage line of the knee joint to the medial malleolus for the deep layer of the intramuscular tendon (Figs. 1 and 2). These locations were identified by confirming movement of muscle fibers in a B-mode ultrasound image during passive movement of the ankle or toes.

The subjects laid on an electrodynamometer (Biodex System 4, Biodex, USA) with the knee in full extension and the ankle joint securely attached to the footplate of the dynamometer. They were instructed to remain relaxed during the measurements. Ultrasound images were recorded along the longitudinal axis of the muscle at ankle DF of 0, 10 and 20°. The examiner checked whether the heel was fixed securely to the footplate of the dynamometer during measurement. The footplate being perpendicular to the fibula was defined as the 0° dorsiflexion of the ankle joint. The same procedure was followed on day 1 and day 2, and the setting of BIODEX was identical on both days. Measurements of each ankle position were performed in the order mentioned, i.e. 0, 10 and 20° DF, to reduce the effect of stretching.

### Statistical analysis

Statistical analyses were performed using statistical software (SPSS Statistics 22, IBM, USA). The intra-class correlation coefficient (ICC) (1.3) using the average value of each day was assessed for the analyses of the interday reliability. In addition, the coefficient of variation (CV) and the standard error of the mean (SEM) were calculated. The CV was calculated by dividing the SD of repeated measurements by the average value, while the SEM was calculated by dividing the SD of repeated measurements by the number of measurements (that is, six times).

A one-way repeated measures analysis of variance (ANOVA) with Bonferroni’s post hoc test were used to determine the differences in the shear elastic moduli between ankle positions. For all tests, the statistical significance was set at *p* < 0.05. On the basis of previous studies [10, 15], the reliability was defined as almost perfect if the ICC was greater than 0.81 and the CV was less than 10%.

## Results

The results of the reliability are shown in Table 1. The ICCs ranged from 0.58 to 0.83 for 0° DF, 0.65 to 0.87 for 10° DF, and 0.71 to 0.95 for 20° DF. The CV ranged from 6.5 to 12.0% for 0° DF, 5.7 to 11.9% for 10° DF, and 4.7 to 13.6% for 20° DF. The SEM ranged from 0.2 to 0.4 kPa for 0° DF, 0.2 to 0.6 kPa for 10° DF, and 0.2 to 1.4 kPa for 20° DF.

**Table 1:** Reliabilities of the shear elastic modulus measurements

	LG	MG	SOL	PL	PB	FHL	FDL	TP s	TP d
DF 0									
Shear elastic modulus of day1 (kPa)	8.7	12.3	4.6	7.0	5.7	4.6	6.0	7.3	7.7
Shear elastic modulus of day2 (kPa)	8.4	11.8	4.7	7.1	5.9	4.9	6.3	7.9	8.0
Mean value of day1 and day2 (kPa)	8.5	12.1	4.6	7.1	5.8	4.8	6.1	7.6	7.8
SD (kPa)	1.7	2.7	1.1	1.7	1.0	1.2	1.4	2.0	1.8
SEM (kPa)	0.2	0.3	0.2	0.2	0.2	0.2	0.2	0.3	0.4
CV (%)	6.5	6.5	12.0	7.6	8.5	9.2	8.4	8.9	11.3
ICC (1.3)	0.77	0.76	0.67	0.80	0.58	0.76	0.78	0.83	0.67
DF 10									
Shear elastic modulus of day1 (kPa)	16.1	25.5	6.4	9.2	7.2	6.0	7.2	8.9	9.3
Shear elastic modulus of day2 (kPa)	15.3	24.8	6.2	8.7	7.4	6.2	7.4	8.7	9.1
Mean value of day1 and day2 (kPa)	15.7	25.1	6.3	8.9	7.3	6.1	7.3	8.8	9.2
SD (kPa)	3.0	5.5	1.5	2.2	1.7	1.5	1.4	2.6	2.6
SEM (kPa)	0.4	0.6	0.3	0.3	0.2	0.2	0.2	0.3	0.4
CV (%)	6.7	5.7	9.6	7.5	8.2	7.0	7.7	8.9	11.9
ICC (1.3)	0.76	0.80	0.80	0.82	0.72	0.87	0.65	0.83	0.76
DF 20									
Shear elastic modulus of day1 (kPa)	31.5	53.7	10.3	11.6	9.5	8.8	9.4	11.4	11.5
Shear elastic modulus of day2 (kPa)	32.2	53.4	10.8	12.3	9.9	8.7	9.4	11.5	11.7
Mean value of day1 and day2 (kPa)	31.8	53.6	10.5	12.0	9.7	8.7	9.4	11.5	11.6
SD (kPa)	6.6	11.5	3.2	4.3	2.8	2.3	2.5	3.9	3.5
SEM (kPa)	0.6	1.4	0.4	0.3	0.3	0.2	0.2	0.3	0.6
CV (%)	4.7	6.3	9.4	7.1	7.9	6.6	6.2	5.6	13.6
ICC (1.3)	0.91	0.82	0.81	0.92	0.85	0.86	0.91	0.95	0.71

*SD* standard deviation, *SEM* standard error in measurement, *CV* coefficient of variation, *ICC* intra-class correlation coefficient, *LG* lateral gastrocnemius, *MG* medial gastrocnemius, *SOL* soleus, *PL* peroneus longus, *PB* peroneus brevis, *FHL* flexor hallucis longus, *FDL* flexor digitorum longus, *TP s* tibialis posterior (superficial layer of intramuscular tendon), *TP d* tibialis posterior (deep layer of intramuscular tendon)

The differences in the shear elastic moduli between ankle positions are summarized in Table 2. The one-way repeated measures ANOVA indicated significant main effects of the ankle position for all muscles. The post hoc analysis showed significant differences between all ankle positions, which shows that the shear elastic modulus increases with the ankle DF for all muscles.

**Table 2:** The differences in the shear elastic moduli between ankle positions

Shear elastic modulus (kPa)			
	DF 0	DF 10	DF 20
LG	8.5 ± 1.7	15.7 ± 3.0	31.8 ± 6.6
MG	12.1 ± 2.7	25.1 ± 5.5	53.6 ± 11.5
SOL	4.6 ± 1.1	6.3 ± 1.5	10.5 ± 3.2
PL	7.1 ± 1.7	8.9 ± 2.2	12.0 ± 4.3
PB	5.8 ± 1.0	7.3 ± 1.7	9.7 ± 2.8
FHL	4.8 ± 1.2	6.1 ± 1.5	8.7 ± 2.3
FDL	6.1 ± 1.4	7.3 ± 1.4	9.4 ± 2.5
TP s	7.6 ± 2.0	8.8 ± 2.6	11.5 ± 3.9
TP d	7.8 ± 1.8	9.2 ± 2.6	11.6 ± 3.5

The data has been expressed as the mean +/− Standard deviation of the values observed on day 1 and day 2. The data has been expressed as the mean of the values observed on day 1 and day 2

## Discussion

This study focused on the ankle plantar flexion muscles and investigated the reliability of shear elastic modulus measurements. To the best of our knowledge, this is the first study examining the shear elastic moduli of the PB, FHL, FDL and TP using shear wave elastography at different ankle positions. The results showed that the CV for the LG, MG and SOL were 4.7 to 6.7%, 5.7 to 6.5% and 9.4 to 12.0%, respectively, which were lower than the CV values of the previous study (9 to 15%, 9%, and 19 to 20%, respectively) [9]. This is probably due to the ankle angle during measurement, which was set more accurately in our study, since it was securely attached to the footplate of the dynamometer.

As for the reliability of the superficial TP, the ICCs were greater than 0.81, and the CVs were less than 10% at all ankle positions, which confirmed the high reliability of the shear elastic modulus measurements of the superficial TP at any ankle positions from 0 to 20° DF. On the other hand, the ICCs of the PL and FHL were less than 0.81, and the CVs were less than 10% at 0° DF, whereas the ICCs were greater than 0.81 at 10° and 20° DF. Furthermore, for the LG, MG, SOL, PB and FDL, the ICCs were greater than 0.81, and the CVs were less than 10% only at 20° DF. These results suggest that high reliability may be attained for shear elastic modulus measurement at 10 and 20° DF for the PL and FHL and at 20° DF for the LG, MG, SOL, PB and FDL.

Our findings suggest that the reliability of the shear elastic modulus measurements differed between ankle positions. The one-way repeated measures ANOVA and post hoc analysis showed that there were significant effects of the ankle positions on the shear elastic modulus for all muscles and that the shear elastic modulus of all muscles increased with ankle DF. These results suggest that the ankle plantar flexion muscles, which were measured in this study, were elongated with ankle DF. The results of this study showed that the ICC at 0° DF was 0.67 for the SOL, which was lower compared to the ICCs of the LG and MG. The previous study had reported that the slack angles (i.e. the joint angle corresponding to the muscle length beyond which the muscle begins to develop a passive force) of the LG, MG and SOL were 14.9 ± 6.7° for plantar flexion, 20.7 ± 6.7° for plantar flexion and 2.0 ± 4.8° for the DF position, respectively [16]. These results suggested that the slack angle of the SOL was greater in the DF direction than those of the LG and MG. Therefore, it is likely that the shear elastic modulus of the SOL measured at 0° did not reflect the subject characteristics. In addition, it was reported that the ICC is affected by the dispersion of the intersubject measured values [17]. It was considered that the low ICC for the SOL was calculated at 0° DF owing to the small SD. For the same reason, lower ICCs may have been calculated for some muscles at 0° or 10° DF.

The ICC for the deep TP was less than 0.81, and the CVs were greater than 10% at any ankle position. The possible reason for the lower ICC and higher CV of the deep TP is the unstable value measured because the shear wave did not reach the target tissue owing to the effect of the superficial tissue (e.g. the SOL, FDL and fascia). On the other hand, the ICCs for the superficial TP were greater than 0.81, and the CVs were less than 10% at all ankle positions. These results suggested that the shear elastic modulus of the TP could be measured more reliably at the proximal superficial layer than at the distal deep layer.

In this study, there was a limitation in that we did not investigate the interrater reliability. Therefore, we were not able to determine whether the shear elastic modulus of a newly measured muscle is comparable among different testers or not. In addition, measurement of the shear elastic modulus also depends on the skill of the tester [18]. Therefore, testers need to practice before taking measurements until a high reliability is obtained, even if a high reliability was shown in this study.

## Conclusions

This study investigated the reliability of shear elastic modulus measurement for the ankle plantar flexion muscles at different ankle positions. The results of this study showed that the measurement reliability of the shear elastic modulus differed between ankle positions and that a high reliability was observed when measuring the shear elastic moduli of the TP at 0 to 20° DF; the PL and FHL at 10 to 20° DF; and the LG, MG, SOL, PB and FDL at 20° DF. These results suggest that the shear elastic modulus of the ankle DF muscle should be measured at 20° DF.

## Abbreviations

ANOVA: Analysis of variance
CV: Coefficient of variation
DF: Dorsiflexion
FDL: Flexor digitorum longus
FHL: Flexor hallucis longus
ICC: Intra-class correlation coefficient
LG: Lateral gastrocnemius
MG: Medial gastrocnemius
MTSS: Medial tibial stress syndrome
PB: Peroneus brevis
PL: Peroneus longus
ROI: Region of interest
SD: Standard deviation
SEM: Standard error of the mean
SOL: Soleus
TP: Tibialis posterior
